# Do Health Beliefs and Behaviors Differ According to Severity of Obesity? A Qualitative Study of Australian Adults

**DOI:** 10.3390/ijerph7020443

**Published:** 2010-02-03

**Authors:** Sophie Lewis, Samantha L. Thomas, R. Warwick Blood, Jim Hyde, David J. Castle, Paul A. Komesaroff

**Affiliations:** 1 Consumer Health Research Group (CHaRGe), Primary Care Research Unit, School of Primary Health Care, Monash University, Building 1, 270 Ferntree Gully Rd, Notting Hill, Victoria 3168, Australia; E-Mail: Samantha.Thomas@med.monash.edu.au; 2 News Research Group, Faculty of Arts and Design, University of Canberra, ACT 2601, Australia; E-Mail: Warwick.Blood@canberra.edu.au; 3 Victorian Department of Health, GPO Box 4047, Melbourne, Victoria 3001, Australia; E-Mail: Jim.Hyde@dhs.vic.gov.au; 4 Faculty of Medicine Nursing and Health Sciences, Deakin University, Victoria 3217, Australia; 5 Department of Psychiatry, St Vincent’s Hospital and University of Melbourne, PO Box 2900, Fitzroy, Victoria 3065, Australia; E-Mail: David.CASTLE@svhm.org.au; 6 Department of Medicine, Monash University, Alfred Hospital, Prahran, Victoria 3181, Australia; E-Mail: Paul.Komesaroff@med.monash.edu.au

**Keywords:** obesity, health beliefs, health behaviors, stigma, public health, qualitative research, consumer perspectives

## Abstract

Public responses to obesity have focused on providing standardized messages and supports to all obese individuals, but there is limited understanding of the impact of these messages on obese adults. This descriptive qualitative study using in-depth interviews and a thematic method of analysis, compares the health beliefs and behaviors of 141 Australian adults with mild to moderate (BMI 30−39.9) and severe (BMI ≥ 40) obesity. Mildly obese individuals felt little need to change their health behaviors or to lose weight for health reasons. Most believed they could “*lose weight*” if they needed to, distanced themselves from the word obesity, and stigmatized those “*fatter*” than themselves. Severely obese individuals felt an urgent need to change their health behaviors, but felt powerless to do so. They blamed themselves for their weight, used stereotypical language to describe their health behaviors, and described being “*at war*” with their bodies. Further research, particularly about the role of stigma and stereotyping, is needed to fully understand the impact of obesity messaging on the health beliefs, behaviors, and wellbeing of obese and severely obese adults.

## 1. Introduction

Obesity is a serious and increasing public health issue associated with a complex range of causes, and health and social consequences [1]. In Australia, it is estimated that about 18% of adults are obese according to their body mass index (BMI ≥ 30) [2]. Of these, about 2% are considered severely obese (BMI ≥ 40) [3]. Whilst those who are severely obese comprise only a small proportion of the obese population, both international and Australian studies show that the prevalence of severe obesity is increasing at nearly twice the rate of obesity overall [3–6]. Individuals who are severely obese experience increased morbidity and mortality from chronic health conditions [7], and incur higher health care costs [8] than those with mild or moderate obesity (BMI 30−39.9).

The health risks associated with obesity have been well documented and include both physical health risks, such as Type II diabetes, heart disease, kidney disease, and some types of cancer [9], as well as emotional health risks, such as depression, anxiety, and low self esteem [10,11]. These risks appear to be highest for individuals classified as severely obese [7,12,13]. The health risk factors associated with obesity are widely promoted by public health agencies, the mass media, obesity researchers, and health professionals. In Australia, public health campaigns have focused on the links between obesity and cancer [14], Type II diabetes [15,16], and reduced life expectancy [17] as a way of motivating individuals to change their lifestyles and lose weight, but also to prevent those who are of normal weight from becoming obese. As yet, there is little evidence to show whether or not these campaigns are having positive or negative effects on rates of obesity in Australia. What is also less clear from current research is how our interventions in the area of obesity may be resonating with the actual experiences of those who are obese. Research from other health issues has shown that understanding social drivers of health beliefs and behaviors are essential in appropriately tailoring public health responses that resonate with populations, communities, and individuals [18]. Consumer perspectives have also been seen as the cornerstones in developing interventions which are translatable to different subgroups within the community.

Obesity research examining health beliefs and behaviors has predominantly focused on whether obese individuals identify that they are obese [19–21], and on the disparities between individual (lay) and expert opinions of the health risks associated with obesity [22,23]. These quantitative studies have concluded that obese individuals may underestimate their weight and that this contributes to the minimization or denial that their weight is a health risk [24,25]. Yet, few studies have examined how severity of weight may impact on health beliefs and behaviors. Whilst health belief literature suggests individuals’ perceptions of their susceptibility and severity of illness can be strong predictors of positive behavior change [26], we are unsure if this applies to severely obese adults. There is limited qualitative research documenting the experiences, opinions and health behaviors of obese individuals. Research that does exist has predominantly clustered all obese individuals together [27,28]. To our knowledge, there are no studies that compare the health beliefs, risk perceptions, and health behaviors of different weight groups within the obese range. Understanding differences between obese and severely obese individuals is important both in tailoring population responses to the obesity epidemic, but also in ensuring that appropriate individual care exists to support behavior change.

## 2. Methods

### 2.1. Approach

The data presented in this paper were part of a more extensive Australian study “*Obesity: Have Your Say”* that explored the experiences, attitudes, and opinions of individuals with a BMI of 30 or more. This study used a qualitative design. Ethical approval was gained from the Monash University Standing Research Ethics Committee.

### 2.2. Sampling and Recruitment

Theoretical, purposive, and strategic sampling methods were used to obtain a representative community sample [29,30]. For example, strategic sampling was utilized to include participants with different attitudes to weight and weight loss, including those who were attempting to lose weight, those who had given up trying to address their weight, and those who were happy with their current weight. A range of different strategies were utilized to recruit participants for this study. These included newspaper and magazine articles, online advertisements, direct recruitment via health professionals, personal trainers and commercial and community-based weight loss centers, and flyers distributed in community areas. The sampling strategy is described in detail in Table 1.

### 2.3. Data Collection

In-depth, semi-structured telephone interviews of between 60 and 90 minutes duration were conducted with participants between April 2008 and March 2009. Interviews were digitally recorded and transcribed within seven days of the interview by a professional transcribing service. The interview schedule included open ended questions related to several broad themes. The data analyzed for this paper focused on three broad themes. (1) Participants’ beliefs about the causes of their obesity; the impact of their weight on their physical and mental health; and the health risks associated with their weight, (2) Participants’ descriptions of previous attempts to change their health behaviors and lose weight, and (3) Participants’ perspectives about key motivators and barriers to behavior change (including weight loss and lifestyle change).

### 2.4. Data Analysis

A descriptive thematic approach was used to analyze the data. Data analysis was conducted by SL and ST. For the purposes of this study, we grouped participants according to standard medical classifications of ‘obese’ (BMI 30−39.9) and ‘morbidly obese’ (BMI 40 and over). The term morbidly obese is often criticized for being stigmatizing and blaming, and so for the purposes of this study we use the terms ‘mild to moderately obese’ (BMI 30−39.9) and ‘severely obese’ (BMI 40 and over). The data were analyzed continuously throughout the study using a constant comparative method. This involved reading and re-reading transcripts, coding and identifying categories/themes (and similarities and differences between these), sorting data to ensure that the concepts/theories were appropriate, and noting similarities and differences between and within the two weight groupings [31,32]. We explored similarities and differences, and sought to understand why differences occurred between the groups. Regular meetings were held to interpret and discuss findings, to develop thematic areas and theoretical concepts, and to refine research questions and identify new research questions as new themes emerged from the data. We tested the reliability of interpretation by randomly selecting 10 transcripts and comparing SL and ST’s interpretation of the data. We then presented these findings for discussion among the broader research team. These meetings were also used to explore any similarities and disagreements in the interpretation of data.

## 3. Results

Quotes are used to represent examples of the research findings. Some responses have also been quantified. We use the term ‘a few’ to refer to less than a quarter of participants; ‘some’ to refer to 25−50% of participants; ‘many’ to refer to 50−75% of participants; and ‘most’ to refer to over 75% of participants. This is to highlight more clearly the proportions of participants who responded in certain ways.

### 3.1. General Characteristics

A total of 172 participants enquired about this study. Of these, 141 participants were included in the study. 23 individuals were excluded from the study because their BMI was less than 30, they lived outside of Australia, or did not reveal their BMI. Eight individuals refused to participate because of the length of the interview or because they thought that the study was testing a new weight loss intervention. Participants’ characteristics are outlined in Table 2. Most were female (n = 105, 74.5%), with an average age of 44.8 years (19−75 years), were married or in a de facto relationship (n = 91, 64.5%), and had a tertiary education (n = 84, 59.6%). About two thirds of participants (n = 88, 62.4%) were classified as obese (BMI 30.0−39.9) and the remainder (n = 53, 37.6%) were classified as severely obese (BMI ≥ 40).

Individuals’ narratives were complex and at times contradictory. Whilst this study sought to understand, and compare risk perceptions, health beliefs and health behaviors between obese and severely obese participants there were extremes of experience within each group. For example, some obese participants reported similar experiences to those who were severely obese, and vice versa. We have summarized the key similarities and differences between the groups in Table 3.

### 3.2. Beliefs about Obesity and Associated Health Risks

Severely obese participants had a sense of resignation about the impact of their weight on their future health outcomes. Most believed that their weight would inevitably lead to additional health problems and shortened life expectancy, and described feeling “*worried*” and “*scared*” about the potential health consequences of their weight:

“*It’s going to definitely impact on my health, it has to really. I know that I’ll be unwell because of my weight.* [But I’ve] *found it really difficult to lose it and then I get more and more concerned.”*

(Female, aged 50, BMI 45.7)

Many also believed that their weight problem was so severe that they would never be able to lose weight:

*“I really to be honest can’t see myself losing* [weight]*. It makes* [me] *feel defeated.”*

(Male, aged 49, BMI 44.1)

Some severely obese participants spoke openly about dying—with an almost fatalistic acceptance of obesity-related mortality:

“There are some changes that have started taking place in my body and there are things that I am aware of, but some days things happen and I just think, oh well, what the hell, if I die, I die.”

(Female, aged 58, BMI 48.9)

Others used dramatic metaphors to describe the impact of their obesity on their mortality—“*walking time bomb*”, “*death sentence*” and “*end up in an early grave*”—suggesting that they believed that their excess weight could kill them without any warning. Many participants believed that the damage they had done to their health by “*gaining so much weight*” was irreversible and that their health would not “*get back to normal*” even if they lost weight:

*“It means I’m losing. I’m watching the time tick on my life and I’m losing the quality of life that I should be enjoying right now. It’s not something you can recover. It is irreversible loss*”.

(Male, aged 61, BMI 44.4)

However, there were exceptions. Those participants who had been obese since childhood described themselves as ‘fat survivors’ because they had avoided or overcome obesity-related illness. Many of these participants attributed their weight to genetic rather than lifestyle factors, and spoke about how their bodies had been resilient to their weight. One woman explained:

“*I know there’s a lot of other physical things that I could be doing, but I suppose I’m just used to carrying this much weight and doctors told me when I was 15 ½ that I should be dead because of my weight and because of my asthma. Well, I’m approaching very rapidly 59 and I’m still here.”*

(Female, aged 58, BMI 48.9)

She goes on to say:

“*Considering my weight and everything I’ve been through medically, hips and arthritis and various operations, medical procedures and things like that, I think I’m a pretty fit person.*”

The language of ‘survivorship’ allowed these participants to frame their obesity experience in a positive way—that instead of obesity being a sign of shame and personal misbehavior, it represented triumph over health adversity.

Obese adults had very different health beliefs and risk perceptions to those in the severely obese group. They believed that although there were health risks associated with obesity, they still had the ability to change their lifestyles to address their weight. However, they believed that their weight did not present an immediate *“threat”* to their health, and as such there was no need to immediately change their health behaviors. This belief was particularly common among obese men, who stated that whilst they agreed that their health may benefit from weight loss, they felt little urgency to change their lifestyles:

“They all say I should lose weight. I agree I should lose weight as well, but I haven’t got a burning desire to lose weight and I don’t think I’ve got a burning need to lose weight, let’s put it that way.”

(Male, aged 66, BMI 33.6)

Few participants in the obese weight range described themselves as ‘obese’. Rather, many used lay language such as “*technically overweight*”, “*labeled as overweight*” or “*fat*” to distance themselves from the negative health and social connotations associated with the word ‘obesity’, and the medicalization of weight:

“*I hate the word ‘obesity’. I think it’s* [part] *of a whole range of negative medical terminology that I think adds to the feelings of helplessness of people who are big...it was never probably the intention of the word but it holds a lot of emotional impact, and I actually do find that quite distressing whenever I hear the word ‘obesity’*.

(Male, aged 42, BMI 35.8)

Others stated that although they were categorized as obese, they did not feel ‘obese’. Obese individuals also tried to distance themselves from individuals they thought were much fatter than themselves:

“I wouldn’t call myself obese because that’s just really a bit of a harsh word because I don’t look very big. I’ve got a friend who’s very obese. I would call her obese but I wouldn’t call myself obese. I’d call myself just fat and overweight and unhappy with myself.”

(Female, aged 34, BMI 38.9)

This differed from those who were severely obese who described themselves in stereotypical ways including: “*lazy*”, “*greedy*”, “*stupid*”, “*unmotivated*” and “*undisciplined*”. Most of these participants believed that their obesity was their own fault, and stated that they blamed themselves for “*wasted*”, “*unfulfilled*” and “*half lives*”:

“I am sad, I am regretful and I feel inadequate that I can’t seem to do anything about it or I haven’t got the discipline to do anything about it, but I know what I should be doing about it. I am an expert on weight loss I think, but I just can’t do it.”

(Male, aged 49, BMI 44.1)

In general, the higher a participant’s BMI, the more they believed that they were to blame for their obesity, and the more they felt that they deserved the health consequences of their health misbehaviors:

“I’ve got all these excuses and I cop out all the time. I’m a failure. And I’m wasting my life.”

(Female, aged 37, BMI 49.4)

There were also key differences in the way obesity impacted on individuals overall lives. Whilst obese participants believed that their weight was the only negative part of otherwise happy and successful lives, severely obese participants believed that their weight controlled and *“consumed”* every aspect of their lives:

“If I’m going to get down about anything, it’s because of the fact that I have a problem losing weight, that’s the one thing in my life that bugs me.”

(Female, aged 74, BMI 33.1)

*“I wake up with it, I sleep with it, I dream of it and it is 24 hours, right around. If I am on the tram coming home, I don’t have anyone sitting next to me.* [Starts crying]*. No one sits next to me on the tram, you know and when I walk in to get up the steps on the tram, people look at me because I have difficulty getting up. It is just so horrible being fat. I don’t want another person to be in the situation that I’m in—it is scary because you don’t know if you are going to wake up the next day.”*

(Female, aged 41, BMI 71.7)

There were also differences in the reasons why participants from the two groups believed they were overweight. Whilst obese participants accepted some personal responsibility for their health behaviors, many believed that social and environmental pressures had played a role in their weight gain—for example their lack of work life balance, their sedentary occupations, their inability to afford healthy foods, and their lack of accessibility or ability to go to a gym:

“*I was busy. I was really worked off my feet for six months and in that six months I put on 20 kilos. I was tired. I was lacking time to prepare healthy meals. I was eating on the run. Plus the stress factor, I was eating to relieve the stress. And I noticed that I was looking for high fat high sugar to keep me going.”*

(Female, aged 51, BMI 38.7)

In contrast, participants in the severely obese weight range often blamed themselves for their obesity. These beliefs also made them feel more *“out of control”* with their weight:

“I’ve always been a bit overweight. As it’s gotten worse and worse I’ve sort of got to the point where I have said ‘well I’m this far gone who cares anymore’ sort of thing, you know it just keeps piling on.”

(Female, aged 21, BMI 41.8)

Severely obese participants also believed that they were trapped by their weight, using words such as “*paralyzed*”, “*imprisoned*”, “*crippled*” and “*turned to stone*”. One man believed that his steady weight gain had *“taken on a life of its own”* after his BMI reached 40, suggesting that the heavier he had become, the more out of control he felt. Others used military metaphors such as *“war”* and *“battle”* to describe their relationship with their weight.

There were however, commonalities between the two groups. The most prominent were descriptions about their frustrations at not being able to lose weight. This was also one of the key contradictions in obese participants’ narratives. Whilst most obese participants felt that they did not need to lose weight and felt healthy, most also described feeling unhappy and frustrated because of previous weight loss attempts:

“*The disappointment, the constant disappointment of giving your best effort to losing weight and it not being successful* [is] *really difficult.”*

(Female, aged 41, BMI 33.5)

This may suggest that despite participants saying that they did not need to lose weight for their health and wellbeing, the social pressures to be thinner still encouraged repeat weight loss attempts.

### 3.3. Health Behaviors

Whilst almost all participants felt a sense of personal responsibility to lose weight, only those in the obese group felt that they could realistically change their lifestyles, and improve their health and wellbeing. Many of these participants stated that they were motivated to *“lose weight”,* and believed that they could do so through diet and exercise. Some stated that their goal was to become “*normal*”, again highlighting the social pressure on participants to be thinner:

“Even though my BMI is high and I’m considered obese, I know with exercise and a good healthy living or a healthy diet, I can get back to normal again.”

(Female, aged 47, BMI 34.0)

Obese participants who were currently trying to change their health behaviors stated that the process of accepting responsibility for their overweight was an empowering experience:

“Something just clicked in my head and something went ‘you keep talking about weight but who’s responsible for that, you are’. It was just this sudden ownership of my weight.”

(Female, aged 37, BMI 31.0)

However, severely obese participants often stated that their weight loss attempts were disempowering. Many stated that as their weight increased it became extremely difficult for them to make changes to their lifestyles, including engaging in physical activity or changing their eating behaviors. These participants stated that on reflection, it would have been easier for them to change their lifestyles when they were less heavy. The heavier they became, the more complex it was for them to change their behaviors:

“When you are really fat it does move into a different level—the physical problems are harder, the consequences are more likely to fall on your head as far as health goes. There tends to be a more complicated situation going on.”

(Female, aged 58, BMI 50.7)

Many also commented that the *“fatter”* they had become, the more disempowered they felt about their health and wellbeing. This was compounded by the advice they received about weight loss from health professionals which lead them to feel “*scared*”, “*daunted*”, “*overwhelmed*” and “*defeated*”. Many commented that when doctors described the amount of weight they would have to lose to become ‘healthy’ and focused attention on weight loss, they felt even more powerless to change their behavior. This was partly because they felt there was limited help and support to make behavior changes—with many describing feeling isolated and “*alone*” in their effort to lose weight:

“It can have that negative impact of overwhelming you and making you feel hopeless and sort of depressing you even further. And just makes you feel guiltier I think when you’re fat I don’t know I just really find the medical profession make you feel bad about yourself.”

(Female, aged 39, BMI 41.1)

Some commented that whilst they had sought regular support from health professionals, the lack of training and simplistic assumptions made by health professionals had added to their inability to change their health behaviors. For example, a common complaint from both obese and severely obese participants was that health professionals made assumptions that weight loss was simply about eating less and exercising more:

“*People don’t need to be told ‘you’re not eating the right stuff, you’re a big fat pig’ and that’s it.”*

(Female, aged 46, BMI 48.8)

As such, when participants tried to take personal responsibility and act upon key messages from their health professionals and public health campaigns to change their behaviors, they felt blamed and unsupported by a health system which was not appropriately equipped to address their complex needs, and provide long term, accessible and affordable support. This led participants to turn to commercial weight loss supports, such as diet companies, which they perceived would be helpful, but ultimately were not sustainable and did not help them to change their lifestyles:

“Jenny Craig, Gloria Marshall, Atkins diet...I’ve been on every diet that you can imagine. I’ve lost weight and I’m now the heaviest I’ve ever been in my life.”

(Male, aged 55, BMI 60.7)

Whilst both obese and severely obese participants described the emotional and physical consequences of failing to change their health behaviors, obese participants were still hopeful that they could change their behaviors, whereas severely obese participants had *“given up”*. Severely obese participants explained how with every failed attempt to change their lifestyle, they felt more disheartened, disempowered and self blaming. Each failed attempt reinforced their lack of self belief in their ability to change and this often prevented them from trying to lose weight again:

“*I just think ‘*[it’s] *too much’, ‘I won’t be able to’. I might be able to do the first 20kg or something, but there’s no way I’ll get any further, so why bother, because I can’t stand the failure again...it would be too disappointing. And it would make me worse.”*

(Female, aged 28, BMI 49.4)

Some stated that their only hope was that science would find a “*magic cure*” for their weight problem:

*“If I live long enough it might be good to hope that they come up with a* [method to] *cut all the fat off or pump a gene into me that munches it all up.”*

(Male, aged 55, BMI 60.7)

These experiences highlight the underlying complexity of the different health behaviors in the two groups, but also the commonalities. Many sought weight loss solutions that were unsustainable in the long term, and were unable to access appropriate care and support.

## 4. Discussion

Before discussing the results, it is important to acknowledge the limitations with this study. The main issue arises with the types of people who agree to participate in these sorts of studies. It is possible that the adults who responded were more likely to be dissatisfied with current approaches to obesity or felt more hopeless about their ability to address their obesity. Secondly, whilst the sample is large for a qualitative study, it was skewed towards older women, and was not reflective of culturally and linguistically diverse groups. As such, the findings of the study cannot be generalized to all individuals living with obesity in Australia. Thirdly, as with any qualitative study, the analysis is reflective of the research team’s interpretation of an extremely complicated set of narratives. Whilst there was consistency of interpretation within our interdisciplinary research team, it may be that others with different disciplinary, philosophical or theoretical approaches could interpret the data in different ways. Finally, qualitative studies may raise more questions than they answer. Further quantitative and qualitative research in this area will allow for increased understanding of the complexity of the issues raised in this paper.

The first findings to emerge from this study were associated with the impact of the stigma and social stereotypes associated with obesity on health beliefs and behaviors. Obese participants tried to distance themselves from the social stereotypes associated with obesity, as well as from individuals who they considered ‘fatter’ than themselves. This concurs with other studies which show obese adults dislike the word obesity, and do not like using it in reference to their own weight [33]. In contrast, participants with severe obesity (BMI 40 and over) described themselves as “obese”, used negative stereotypes to describe themselves, and blamed themselves for their obesity. Research to date has been conflicting about whether obese adults stigmatize themselves, and each other. Some studies show that obese individuals stereotype individuals who are fatter than themselves [34], others show that they internalize the stigma [35–37], and some show that they challenge the social stereotypes associated with obesity [37,38]. However, our study suggests that severity of obesity may play a key role in individuals’ perceptions of their weight, their views of other obese individuals, and their beliefs about the causes of their obesity.

Severity of obesity also influenced individuals’ perceptions about their personal health risk. As in other studies [22,23,25], obese participants in this study did not perceive that their weight was a risk to their health. This was a particularly common experience for men in our study. Whilst obese participants felt empowered to, and capable of changing their health behaviors, they did not feel that there were any immediate health reasons why their behavior had to change. Rather, weight loss attempts were motivated by social pressures. As such, there may be a disconnect between messages encouraging people to lose weight for health reasons, and the actual drivers of weight loss behaviors. In contrast, whilst severely obese participants felt a sense of urgency in trying to change their health outcomes, they felt unable to change their health behaviors. For these participants it was a case of *“too little, too late”*. They were also more critical about the lack of support and help from health professionals in addressing their obesity. Further research should examine the complexity of the relationship between severely obese individuals and their health professionals to ascertain why there may be a disconnect around weight and weight management [39–41]. One hypothesis may be that there are many miscommunications and misunderstandings between obese individuals’ experiences and beliefs about their weight and the reasons for their behaviors, and the perceptions and beliefs of health professionals. For example, doctors may blame obese patients for looking for an ‘easy way out’ of their weight problem [42,43]. Severely obese participants in this study were looking for a ‘magic cure’ for their obesity, but this was because they had given up on themselves and their own ability to lose weight.

What is less clear from this study is the impact that the current public focus on obesity may have on obese individuals. Individuals in the obese weight range appear to take up messages about the social and environmental factors associated with obesity but are distancing themselves from health risk messages and the medicalization of ‘being fat’. Conversely, severely obese individuals in this study internalized stereotypical messages and equated the severity of their obesity with severe health outcomes. It was interesting that severely obese participants described themselves as ‘at war’ with their weight, similar to the military metaphors present in much of the public discussions of the ‘war on obesity’ [44]. Over twenty years ago, Susan Sontag described that the use of war metaphors in health inevitably leads to blame being attributed to the individual, rather than focusing on the illness itself [45]. The next step in research in this area should be to understand whether our public health and policy responses to obesity may be stigmatizing and disempowering some groups of obese individuals, and may negatively influence their health beliefs, behaviors and outcomes.

## 5. Conclusion

This study suggests that the health beliefs and behaviors of obese individuals may vary according to the severity of their obesity. Further understanding of the socio-cultural and individual factors associated with these beliefs and behaviors is important in developing population-based messages which resonate with different groups of individuals, but also in developing appropriate interventions for those who wish to change their behaviors. Qualitative research adds a new dimension to our understandings of the complex factors associated with different subgroups of obese individuals.

## Figures and Tables

**Table 1.** Sampling strategy.

**Sampling method**	**Time frame**	**Reason to include this strategy**	**Respondents**	**Excluded**	**Participants**
Stream One ➢ Database of obese individuals willing to participate in research ➢ University and workplace mailing lists ➢ Electronic advertisements ➢ Study website	April–October 2008	Initial convenience sample	n = 59	n = 12	n = 47
Stream Two ➢ Article in the Herald Sun (Victoria’s most read newspaper) appeared on April 26^th^ 2008	April–July 2008	To diversify the sample to include individuals from a broader range of socio-economic groupings	n = 28	n = 1	n = 27
Stream Three ➢ Article in the Leader (community newspaper) ➢ Article in Good Health and Arthritis magazines ➢ Direct recruitment through health professionals (e.g., clinicians, dietitians) ➢ Posters and flyers left in community areas (e.g., plus size clothing stores, shopping centers) and obesity and weight loss centers or groups (e.g., Weight Watchers, Overeaters Anonymous) ➢ Internet message boards, forums and groups	May 2008–March 2009	To recruit participants from local community areas and groups; those living outside Victoria; and those who may not access mainstream mass media	n = 32	n = 6	n = 26
Stream Four ➢ Posters and flyers left at gyms and recreational facilities (e.g., Curves, Contours, Fitness First, Fernwood) ➢ Direct recruitment through personal trainers	July–September 2008	To include participants who are engaged in physical activity	n = 4	n = 0	n = 4
Stream Five ➢ Workplace, hospital, and university mailing lists and advertisements asking for participants who are overweight and male	October 2008–March 2009	To recruit men, people who do not consider themselves obese, and people with lower BMI (30–35)	n = 41	n = 11	n = 30
Stream Six ➢ Snowball recruitment (e.g., friends, family, work colleagues)	April 2008–March 2009	Convenience sample	n = 8	n = 1	n = 7

**Table 2.** Participant demographics.

	**Total**	**Obese participants**	**Severely obese participants**

**Demographic category**	**n(%)**	**n(%)**	**n(%)**

Total	141	88(62.4)	53(37.6)

**Gender**			
Female	105(74.5)	60	45(84.9)
Male	36(25.5)	28	8(15.1)

**Age**			
Mean	44.8	45.5	43.6
Range	19–75	19−74	21−75

**BMI**			
Mean	39.3	34.6	47.1
Range	30.0−71.7	30.0−39.9	40.3−71.7

**Marital status**			
Single	50(35.5)	25(28.4)	25(47.2)
Married/De facto	91(64.5)	63(71.6)	28(52.8)

**Education**			
No formal qualification	15(10.6)	7(8.0)	8(15.1)
Secondary school graduate	19(13.5)	14(15.9)	5(9.4)
Vocational training	23(16.3)	15(17.0)	8(15.1)
Completed undergraduate	45(31.9)	26(29.5)	19(35.8)
Completed postgraduate	39(27.7)	26(29.5)	13(24.5)

**Income before tax (AUD)**			
<50,000	48(34.0)	26(29.5)	22(41.5)
50,000−100,000	59(41.8)	42(47.7)	17(32.1)
>100,000	32(22.7)	19(21.6)	13(24.5)
Not revealed	2(1.4)	1(1.1)	1(1.9)

**Table 3.** Commonalities and differences between the health beliefs and behaviors of obese and severely obese participants.

**Differences**	**Obese**	**Severely obese**

*Beliefs about obesity and associated health risks*	Describe themselves as overweight or fat	Describe themselves using medicalized terminology (*i.e*., obese)
	Seek to distance themselves from the stereotypes associated with obese individuals, and those who are heavier than themselves	Describe themselves in stereotypical language of obesity
	Do not believe that their weight is an immediate health risk	Believe that their weight is a serious and immediate health risk
	Believe that their obesity is caused by social and environmental factors	Believe that they are to blame for their obesity
*Health Behaviors*	Feel empowered to change health behavior to address their weight	Feel powerless to change health behavior to address their weight
	Have high expectations that changing health behaviors will lead to positive health outcomes	Have low expectations that changing health behaviors will lead to positive health outcomes
	A sense of personal responsibility to lose weight encourages empowerment	A sense of personal responsibility encourages powerlessness
	Social pressures to be thinner drive motivations to lose weight	Health risks provide the key motivations for weight loss.

**Commonalities**	Believe that they are personally responsible for changing their health behaviors and losing weight	
	Failed attempts to lose weight decreases motivation and empowerment to change health behaviors	
